# Understanding Material Removal Mechanism and Effects of Machining Parameters during EDM of Zirconia-Toughened Alumina Ceramic

**DOI:** 10.3390/mi12010067

**Published:** 2021-01-09

**Authors:** Azat Bilal, Asma Perveen, Didier Talamona, Muhammad Pervej Jahan

**Affiliations:** 1 Department of Mechanical & Aerospace Engineering, Nazarbayev University, Nur-Sultan 010000, Kazakhstan; azat.bilal@nu.edu.kz (A.B.); asma.perveen@nu.edu.kz (A.P.); didier.talamona@nu.edu.kz (D.T.); 2 Department of Mechanical & Manufacturing Engineering, Miami University, Oxford, OH 45056, USA

**Keywords:** ceramics, thermal spalling, taper, circularity, overcut, materials removal rate, material removal mechanism

## Abstract

Non-conductive structural ceramics are receiving ever-increasing attention due to their outstanding physical and mechanical properties and their critical applications in aerospace and biomedical industries. However, conventional mechanical machining seems infeasible for the machining of these superior ceramics due to their extreme brittleness and higher hardness. Electro discharge machining (EDM), well known for its machining of electrically conductive materials irrespective of materials hardness, has emerged as a potential machining technique due to its noncontact nature when complemented with an assistive electrode technique. This paper investigates the material removal mechanism and effects of machining parameters on machining speed and dimensional and profile accuracies of features machined on zirconia toughened alumina (ZTA) ceramics using assistive electrode EDM. Our experimental results demonstrate that both increasing peak current and pulse on time improves the MRR, however, it also aids in generating thicker layer on machined surface. In addition, pulse interval time is crucial for the machining of nonconductive ceramics, as larger value might cause the complete removal of intrinsic carbon layer which may lead to non/sparking condition. Higher peak current increases circularity whereas short pulse on and pulse off time aid in increasing circularity due to rough machining. In addition, taperness is found to be regulated by the peak current and pulse on time. Overall, thermal cracking and spalling appear to be a dominating material removal mechanism other than melting and evaporation for the EDM of ZTA.

## 1. Introduction

Ceramics are considered as one of the outstanding structural materials due their excellent properties such as high hardness, higher strength, superior chemical stability, low density, high temperature strength, high wear resistance, and biocompatibility. Because of these superior characteristics, ceramic materials found their application not only as cutting tools but also in the aerospace and biomedical industries [1]. However, ceramic materials are also well recognized as difficult to machine materials due to their associated enhanced mechanical characteristics. As a result, the conventional machining of ceramics faces challenges such as high cutting forces, higher tool consumption, the generation of microcracks in mechanical machining, and complex and expensive manufacturing cycle specially in the final stage of machining. Thus, the main challenge in achieving wider application of ceramics is to develop further machining techniques which would allow economical machining as well as decreased residual damage [2].

The literature has reported on the several non-conventional techniques such as laser machining, electron beam machining, chemical machining, plasma machining, ultrasonic machining, electrochemical machining, abrasive water jet machining that contributes towards the machining of ceramics. Table 1 shows a brief overview of various techniques for ceramics machining.

Apart from these techniques, another non-traditional machining techniques such as electro discharge machining (EDM) has also demonstrated its potential as an alternative machining technique for ceramics materials, as it offers several attributes including less tool consumption, no cutting forces, non-contact process, and independency of material hardness. This is an electrothermal process which removes material by melting and vaporization and therefore allows the machining of brittle materials irrespective of the mechanical properties such as wear resistance and hardness [27,28]. However, a successful EDM process requires workpiece with certain level of electrical conductivity (10^−2^ Ω^−1^ cm^−1^). Therefore, EDM can be applied to conductive ceramics without any issue, however, for non-conductive ceramics this process seems not feasible. Although electrical conductivity of the non-conductor ceramics can be enhanced to the required minimum level by doping with conductor materials, it has its downside of reduced mechanical properties as well. Therefore, one of the modified EDM approaches known as Assistive Electrode Method allows for the machining of nonconductive ceramics with the help of some metallic conductive layer on the workpiece [29,30,31]. In this assistive electrode approach, the conductive layer initiates EDM process, and then pyrolytic carbon molecules generated from dielectric attach themselves with the nonconductive workpiece and continue the EDM process by providing the necessary electrical conductivity [32,33]. This process is regulated by the generation of this intrinsic carbon layer which is mainly impacted by the machining parameters, dielectric type, and nature of the conductive layers [34,35]. Although several studies reported on the EDM machining of Si_3_N_4_ [36,37], SiC [38,39,40], Al_2_O_3_ [41,42,43] and ZrO_2_ [33,44,45,46] ceramics, investigation related to the EDM machining of zirconia toughened aluminum (ZTA), which has potential applications in orthopedic prostheses, cutting tools, and wear-resistant parts, is inadequate. Therefore, this study focuses on EDM machining of ZTA ceramics. In the earlier investigation [47], the authors have thoroughly investigated the coating mechanism for machining of nonconductive ceramics. However, it is also equally paramount to corelate the input machining parameters and performance parameters in order to identify the optimized parameters for desired outcomes. Although the Die Sinking EDM machine can offer the variations for some input parameters such as current, pulse on time, and pulse off time, it is also imperative to identify those most important parameters among all input parameters, which can impact the machining of materials. This paper intends to explore material removal mechanisms and present the parametric study of zirconia toughened aluminum where four performance parameters such as materials removal rate, overcut, circularity error, and taper were investigated for three different variation of current, pulse on time, and pulse off time using assistive electrode Die Sinking EDM. Three level full factorial design was used for designing the experiments to evaluate both main effects as well as interaction effects among all input parameters. Twenty-seven sets of experimental trials were planned as per this full factorial design to evaluate the main parameters effect and interactive effect.

## 2. Experimental Details

Experiments on Zirconia-toughened Alumina (ZTA) ceramic workpieces were carried out by using Excetek ED400 die-sinker EDM machine. The successful machining of non-conductive ceramics using a newly developed Assisting Electrode method was achieved. As an assisting electrode, an electrically conductive coating consisting of three different layers was used. The bottom layer of the coating was silver paint applied by brush, the middle layer was carbon nanofibers, and the top layer was copper tape. Ceramics with applied coating was baked for an hour at 250 °C for the coating to combine with the ceramic surface. For a successful EDM of electrically non-conductive ceramics, one of the crucial steps was applying this conductive coating. The thickness of each layer in the coating affects the stability of machining. Stable machining mainly depends on the amount of carbon nanoparticles and its uniformity on the surface. The preparation time for the new coating was an hour and the experimental results suggest its effectiveness. The assistive electrode proposed in this study is different from those reported in literature and is prepared after initial investigative study with trial and error, as demonstrated in our previous studies and its thickness is 300 µm roughly [47,48].

**Figure 1:**
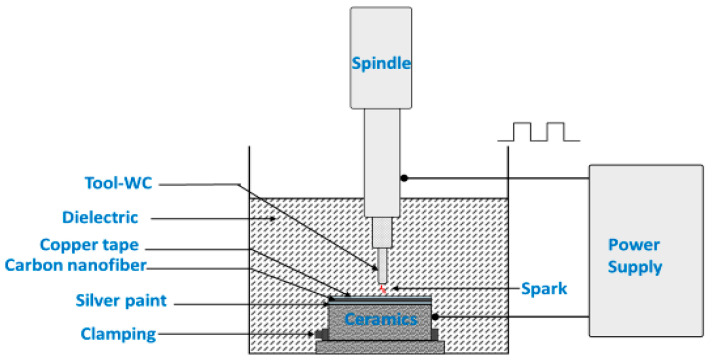
Schematics of experimental set-up.

The most commonly used experimental designs involved in manufacturing industries use full and fractional factorial designs at 2-levels or 3-levels. The joint and individual effect of every factors on the desired response parameters can be observed using factorial designs where the factorial design can be either full or fractional factorial [49]. The 3^k^ design is obviously a prospective alternative for a researcher who has concerned about curvature in the response function [50]. A three level full factorial design was used for designing the experiments to evaluate both main effects as well as interaction effects among all input parameters. Here, we have chosen three levels for three input parameters, because one of the assumptions we make for factors at 2-levels is that the response is approximately linear over the range of the factor settings chosen which is not the case for our study clearly [49]. During the experiments, 27-hole features were created on ZTA ceramic workpieces with dimensions of 20 mm × 20 mm × 3 mm. The tool used was rod type Tungsten Carbide (WC 92%, Cobalt 8%) with a diameter of 1.9 mm. This study shows the effectiveness of proposed assistive electrode technique by demonstrating the machining through holes. However, in order to investigate material removal mechanism and the migration of material and machined surface composition, blind holes were machined and analyzed. Figure 1 shows schematic representation of the EDM machine that was used in this study. Table 2 provides the details of ZTA workpiece and Table 3 demonstrates the details of the Die-Sinking EDM machine used in experiments. The design of experiments (DOE) and respective measurements of machining performance parameters are shown in Table 4.

Three different important machining parameters at three levels were applied. Those are Discharge Current (1 A, 3 A, and 5 A), pulse on time (100 µs, 150 µs, and 200 µs), and pulse off time (200 µs, 250 µs, and 300 µs). In this study, the voltage was kept constant at a comparatively lower settings, as it was reported in authors previous studies that comparatively lower settings of voltage resulted in more effective and successful electro-discharge machining of nonconductive ceramics [51]. Previous studies by [52] have suggested that tool rotation does not assist much in machining ceramics. As such, the tool rotation was turned off for these set of experiments. Hydrocarbon oil was used as dielectric.

In this study, to investigate the material removal mechanism, the machined surfaces were analyzed using scanning electron microscope (SEM) and energy dispersive X-ray spectroscopy (EDS). The EDS mapping of machined surfaces provided locations of various migrated and existing materials on the machined surface, whereas the EDS spectrum analysis revealed the approximate percentages of different materials on the machined surface. The machining performance was analyzed by evaluating the volumetric material removal rate (machining speed) and assessing dimensional and profile accuracies of the machined holes. Equations (1)–(4) were used for the calculation of various performance parameters as shown below. In Equations (1)–(4): MRR = material removal rate (mm^3^/s), d_bottom_ = diameter of the hole at the bottom/exit (mm), d_top_ = diameter of the hole at the top/entrance (mm), h = thickness of the workpiece (mm), d_largest_ = distance between two furthest points on the profile of the hole at the entrance (mm), d_smallest_ = distance between two nearest points on the profile of the hole at the entrance (mm).

(1) $$\mathrm{MRR}=\frac{({\left(\frac{d_{\mathrm{bottom}}}{2}\right)}^{2}+\left(\frac{d_{\mathrm{bottom}}}{2}\right)\left(\frac{d_{\mathrm{top}}}{2}\right)+{\left(\frac{d_{\mathrm{top}}}{2}\right)}^{2})\pi h}{3\times \mathrm{Machining}\;\mathrm{time}}$$

(2) $$\mathrm{Overcut}=\frac{d_{\mathrm{top}}-d_{\mathrm{tool}}}{2}$$

(3) $$\mathrm{Circularity}\;\mathrm{error}=d_{\mathrm{largest}}-d_{\mathrm{smallest}}$$

(4) $$\mathrm{Taper}=\tan^{-1}\left(\frac{d_{\mathrm{top}}-d_{\mathrm{bottom}}}{2h}\right)$$

## 3. Results and Discussion

### 3.1. Analysis of Material Removal Mechanism

**Figure 2:**
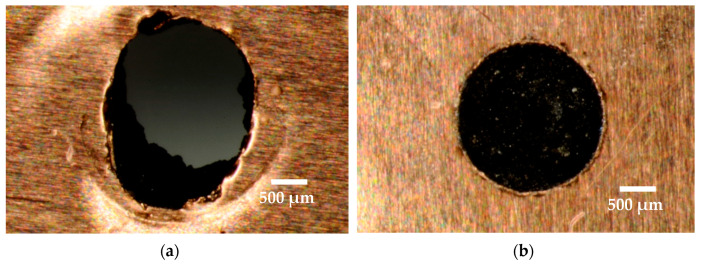
Images from optical microscope: (**a**) Through hole (I_p_ = 5 A, T_on_ = 100 µs, T_off_ = 300 µs); (**b**) Blind hole (I_p_ = 1 A, T_on_ = 200 µs, T_off_ = 250 µs).

In this study, machining was carried out on a non-conductive ZTA workpiece using the specially designed assistive electrode method. The design of the assistive electrode has been discussed in the experiment section. Figure 2 shows an example of through hole and blind hole machined by two different settings of parameters. Although the ZTA ceramic is an electrically non-conductive material, the assisted electrode method resulted in successful through holes, as can be seen in Figure 2a. However, because of very slow material removal rate, blind holes were machined to analyze the surface topography, composition, and migration of materials, and hence obtaining a fundamental understanding of the material removal mechanism during EDM of non-conductive ceramics.

**Figure 3:**
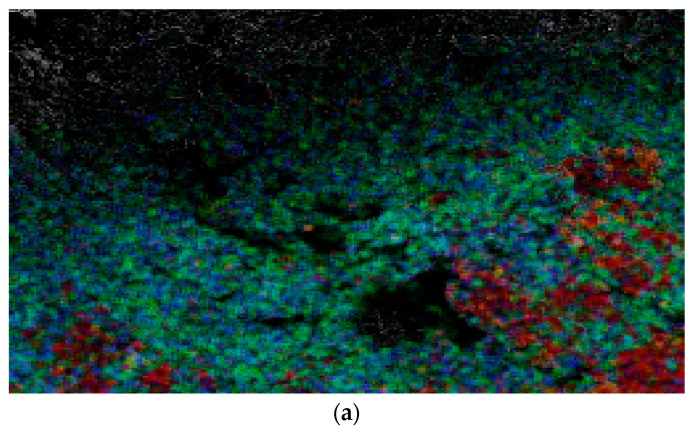

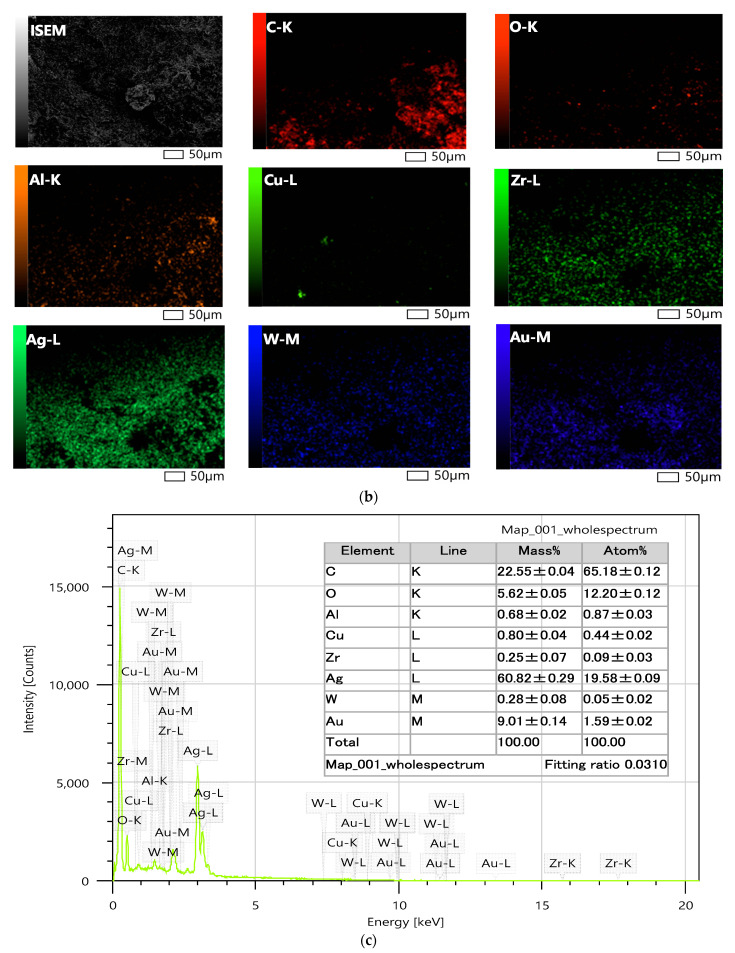
(**a**) Energy dispersive X-ray spectroscopy (EDS) mapping of the machined surface (hole bottom; Exp. #8—Table 3) at machining parameters of 80 V, 1 A, T_on_ = 200 µs, T_off_ = 250 µs, (**b**) mapping of individual element showing the locations and intensity of individual elements, (**c**) EDS spectrum analysis showing percentage of different elements present on the machined surface.

As the EDM of non-conductive ceramics is made possible by the assistive electrode method, the material removal mechanism is somewhat different than the traditional EDM of conductive metals. Surface modification and the migration of material play major roles in progression of machining during the EDM of non-conductive ceramics. The main challenge is the formation of electrically conductive layers where sparking can continue to occur. The sparking starts with the conductive coating in the assistive layer, and then continues on the machined surface due to surface modification and migration of electrically conductive materials from the coating and tool to the workpiece. As can be seen from EDS mapping of the machined surface (bottom of hole) presented in Figure 3 carbon, oxygen, and tungsten migrated to the machined surface. The presence of copper and silver indicates the migration of electrically conductive materials from the assistive electrode. Carbon constitutes a huge portion of materials that were deposited on the machined surface. It comes from two sources, primarily from the carbon nanofibers that was applied as a conductive layer and secondarily from the decomposition of dielectric. The presence of oxygen is due to oxidation and copper and silver come from the conductive layer that was applied on top of ceramics. Aluminum and zirconia originate from the ceramics itself. The migration of tool particles on the surface of ceramics can be observed due to the presence of tungsten. Also, gold can be seen from the elemental analysis table on EDS spectrum, which was applied on the machined surface to make the ZTA ceramic surface electrically conductive for taking SEM images.

**Figure 4:**
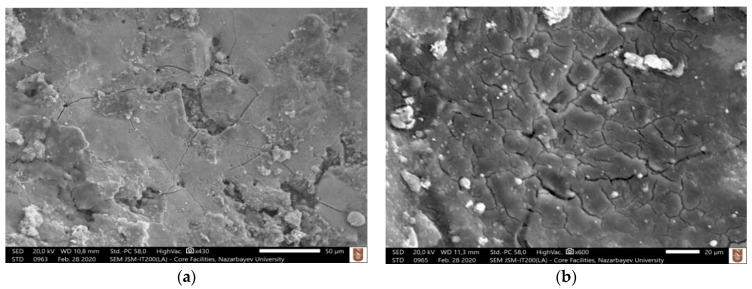

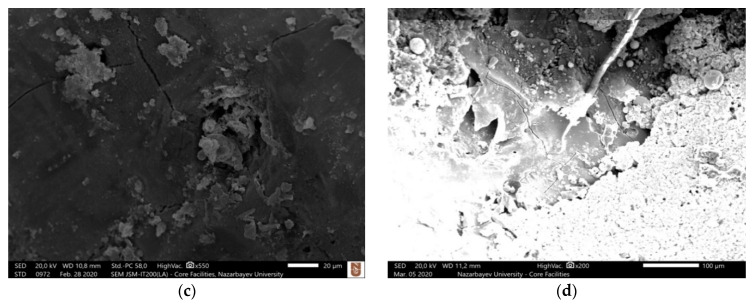
Examples of micro-cracks on the machined surface of blind holes at various combination of parameters; (**a**) Exp. # 1 (Table 3) (80 V, 1 A, T_on_ = 100 µs, T_off_ = 200 µs.), (**b**) Exp. # 2 (80 V, 1 A, T_on_ = 100 µs, T_off_ = 250 µs.), (**c**) Exp. # 5 (80 V, 1 A, T_on_ = 150 µs, T_off_ = 250 µs.), (**d**) Exp 24 (80 V, 3 A, T_on_ = 150 µs, T_off_ = 300 µs.).

**Figure 5:**
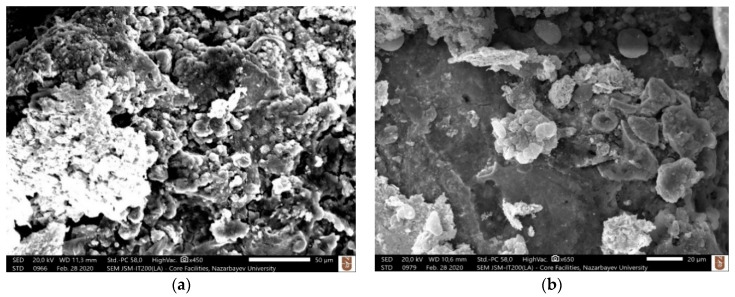

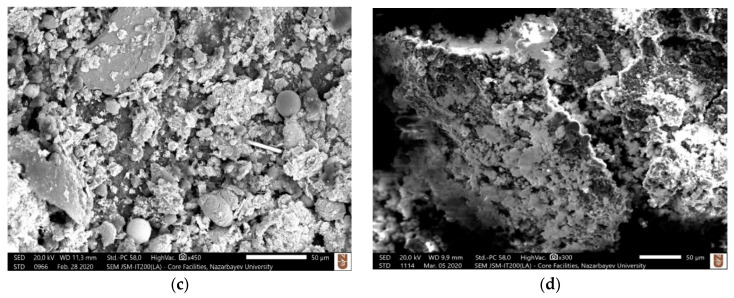
Example of thermal spalling on the machined surface at different experimental conditions, (**a**) Exp 2 (80 V, 1 A, T_on_ = 100 µs, T_off_ = 250 µs.), (**b**) Exp 7 (80 V, 1 A, T_on_ = 200 µs, T_off_ = 200 µs.), (**c**) Exp 9 (80 V, 1 A, T_on_ = 200 µs, T_off_ = 300 µs.), (**d**) Exp 20 (80 V, 3 A, T_on_ = 100 µs, T_off_ = 250 µs.).

**Figure 6:**
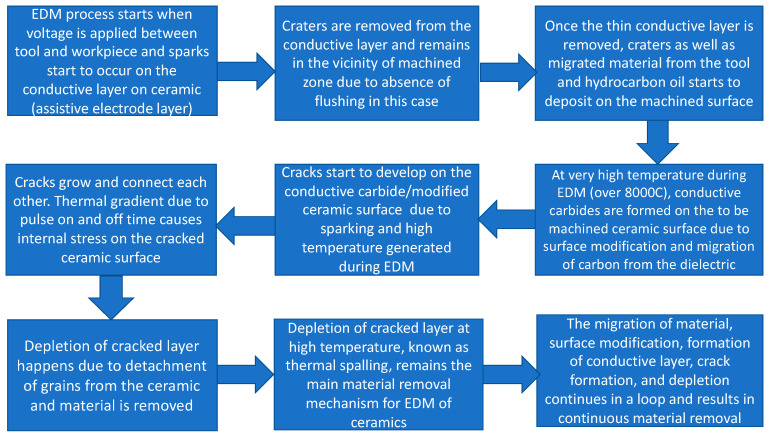
Step-by-step material removal mechanism during EDM of electrically non-conductive ceramic.

As a result of the migration of materials and due to high temperature generated during machining, it was hypothesized that conductive metal carbides are formed on the machined surface, which cracks and peels off the machined surface due to the continuous sparking and thus facilitates the material removal. It is reported in the literature that the material removal mechanism of ceramics machined by assistive electrode method can be characterized by five distinguished phenomena, melting and evaporation, thermal spalling, decomposition and oxidation, fusion and vaporization, and electromagnetic and electrostatic force [53]. In this study, some of these characteristics were observed. The migration of material discussed in the previous section is an indicative of the decomposition and oxidation mechanism. The most common mechanism of material removal observed during the EDM of non-conductive ZTA using assistive electrode method in this study was thermal spalling. Thermal spalling, typically seen in the refractory materials, includes breaking, cracking, and then finally peeling off layers due to high temperature. Due to the higher temperature (above 8000 °C) involved in the EDM process, thermal spalling is considered as one of the associated material removal mechanism where grain/s of material is peeled off from a large body. This occurs as a result of thermal impact due to the higher EDM process temperature and leads to ultimate mechanical breakdown. During the process, thermal gradient induces internal stress, which in turn causes the initiation and propagation of micro-cracks. Consequently, tension or shear failure occurs due to increased thermal extension force. In case of ceramics materials, spalling may cause the detachment of both single and multiples grains. Intergranular cracks and/or transgranular crack contribute towards the materials removal. Higher stress contact point is favored for thermal spalling [54]. For almost all cases of machined blind holes at different combinations of machining parameters, cracks were observed on the machined surface. Figure 4 shows examples of machined surfaces with thermal cracks on the surface. Table 5 presents the measurements of lengths of three to five micro-cracks on the machined surfaces shown in Figure 4. It can be seen that the average length of micro-cracks increases with the increase of pulse on time and pulse off time. It is also found that the cracks are interconnected, which facilitate the material removal during the thermal spalling process. Thermal cracking is the first step of the thermal spalling process, which leads to eventual peeling off of the layers or materials from the ceramic surface. Thermal spalling leads to a very rough and irregular surface of the machined ceramic, as can be seen from examples provided in Figure 5. It is important to note that thermal spalling on machined ceramic surface exhibits much rougher and irregular surface than traditional EDM, thus distinguishing thermal spalling process of material removal mechanism compared to typical EDM machined surface. The random deposition of molten debris on the machined surface found in all the images indicates the melting and evaporation mechanism of material removal, which is an obvious material removal mechanism for any EDM process. The step-by-step mechanism of material removal during the EDM of electrically non-conductive ceramics (zirconia toughened alumina (ZTA) for this study) is presented in Figure 6.

### 3.2. Analysis of Material Removal Rate (MRR)

**Figure 7:**
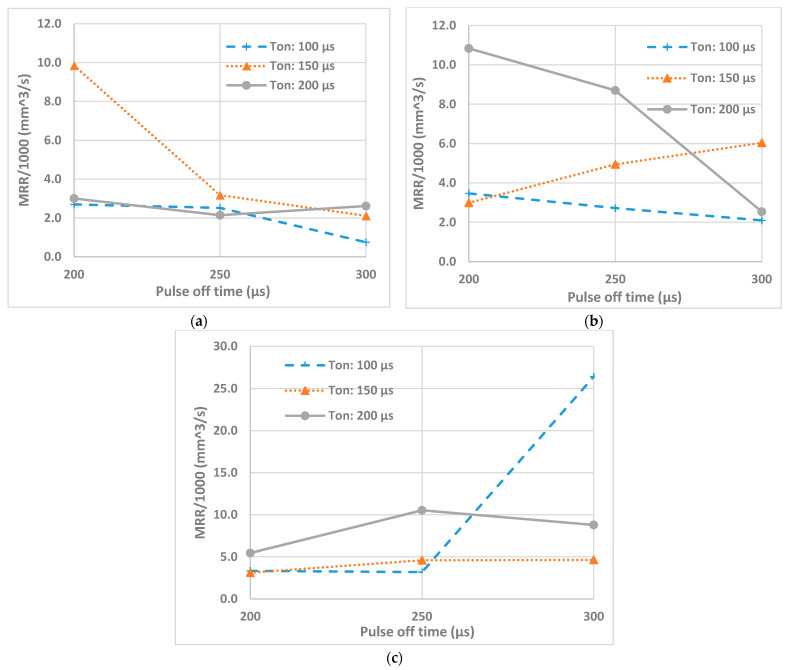
Effects of T_on_ and T_off_ on material removal rate (MRR) at Peak Current (**a**) 1 A, (**b**) 3 A, (**c**) 5 A.

Figure 7 shows the effects of pulse on time and pulse off time at different peak currents on the MRR. As it can be seen from Figure 7, for machining at 1 A peak current, increasing the pulse off time at pulse on time of 150 µs leads to a rapid decrease of MRR. For the machining at 3 A, rapid decrease of MRR can be observed at pulse on time of 200 µs. Except for a few occasions, the MRR decreased with the increase of pulse off time or pulse interval. This is due to the fact that with the increase of pulse interval the duty ratio decreases, i.e., the percent of time the current is passing through is reduced, which results in lower amount of material removal per unit of time. Although higher settings of the pulse off time decreases MRR, there need to be an optimal setting of pulse off time in order to ensure stable machining operation by reducing number of arcing and short-circuiting pulses [55].

**Figure 8:**
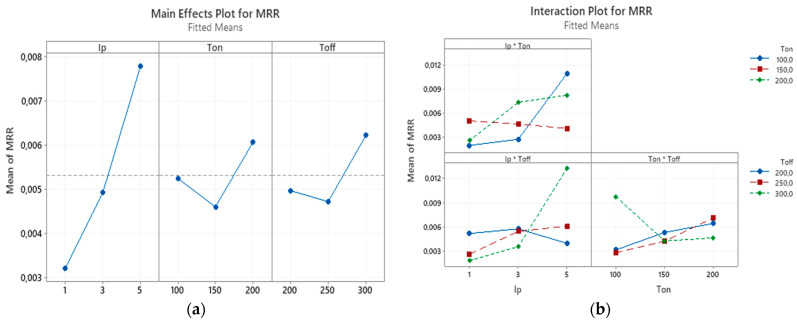
(**a**) The main effects plot and (**b**) the interaction effects plot for the MRR.

For efficient machining operation and higher MRR, the optimum parameter of pulse off time is important since the machined debris needs to be removed. If debris are not removed, they melt and reattach to the machined surface leading to inefficient discharges, which in turn lowers MRR. The interaction effects and main effects of process parameters of EDM of nonconductive ZTA on MRR were studied by conducting variance analysis by plotting these effects. In the Figure 8a the main effect plot for the MRR can be observed. It can be seen from Figure 8a that none of the trend lines are horizontal or parallel to the *X*-axis, which means the main effect for all three cases are present. It can also be noted that the slopes of all three trend lines are steeper indicating greater magnitude of the effect of peak current, pulse duration and pulse interval on the MRR. For each parameter level, the means of MRR can be seen on this plot. Based on this plot, increasing the peak current raised the MRR. The increase in current resulted in the increase of discharge energy, which in turn raised MRR. For pulse on time and pulse off time the trend is similar, with the increase from −1 level to 0 level, MRR decreases, however the increase from 0 level to +1 level leads to the increase in MRR. But overall, these fluctuations are not significant enough. Similarly, higher values of pulse on time and pulse off time (to make sure complete removal of debris) are desired for maximum values of MRR for usual conductive materials. However, center points have a lower value of MRR than both the corner points. This shows that the relation between the response variable MRR and T_on_, T_off_ is not linear. Increased T_on_ can cause higher MRR but also can cause redeposition of thick layer, thus results in reduced MRR. Whereas increased T_off_ also can cause the total removal of debris from the workpiece surface and therefore machining may not continue further due to the lack of intrinsic carbon layer presence. T_off_ value is critical for machining of nonconductive materials, as complete debris removal is unwanted.

Figure 8b shows the interaction effect plot for the MRR. During the interaction plot, parallel lines indicate no significant interaction, whereas non-parallel lines indicate the existence of interaction between the parameters. From the interaction plot it can be observed that the interaction effects are present for different combination of peak current, pulse on time, and pulse off time on the MRR. The most significant interaction effect for MRR among these three is between peak current and pulse off time. It can be observed that the interaction effects between peak current and pulse on time and the interaction effect between pulse off time and peak current are minimal. Generally, the MRR increases with combined increase of peak current and pulse on time. The MRR was also increased with the increase of peak current, as with the increase of peak current the discharge energy per pulse increases resulting in larger crater (unit volume of material removed per pulse) [56].

### 3.3. Analysis of Dimensional and Profile Accuracy

#### 3.3.1. Analysis of Overcut

**Figure 9:**
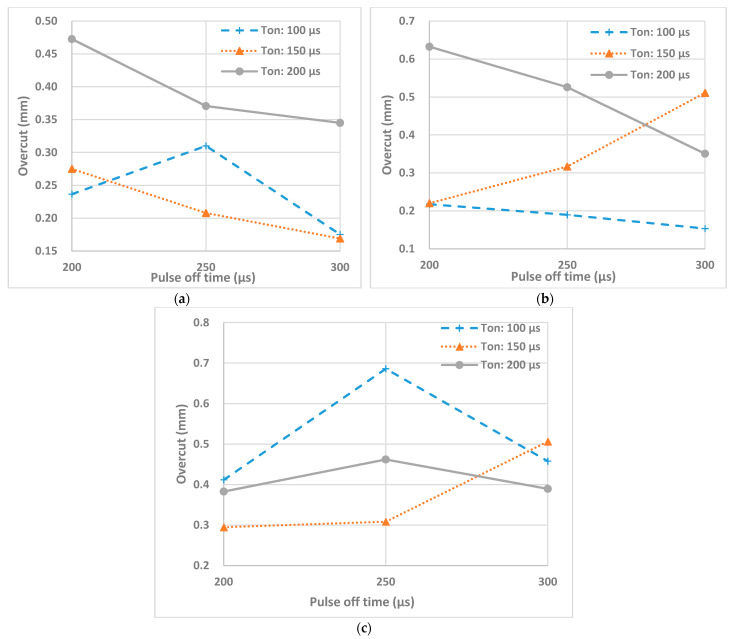
Effects of T_on_ and T_off_ on overcut at peak currents (**a**) 1 A, (**b**) 3 A, (**c**) 5 A.

Figure 9 shows the effects of T_on_ and T_off_ at different peak currents on the overcut of the machined holes on ZTA ceramic workpiece. Based on the Figure 9, increasing pulse off time generally lead to decrease of overcut. This is due to the fact that increasing the pulse interval helps flushing out of debris from the machining zone, thus reducing the attachment of debris around the edge of the micro-holes. The attached debris around the holes causes secondary sparking at the rim of holes, thus enlarging the overcut of the holes [57]. However, as discussed earlier, the pulse interval itself does not define the machining performance, as the performance also depends on combined effect of pulse duration and pulse interval. It is found that there are cases with the increase of overcut for increasing pulse off time at pulse on time of 150 µs and peak currents of 3 A and 5 A. As a result, it can be said that optimal combination of pulse on time and pulse off time where the value of pulse off time is high enough results in lower overcut. It is also found that for a specific setting of pulse on time and pulse off time, the overcut increases with the increase of peak current. It can be seen that except for a few discrepancies, the values of overcut are slightly higher for Figure 9c, when a peak current of 5 A was used. As the peak current increases, the crater size becomes broader, thus increasing both the MRR and overcut of the machined holes [55].

**Figure 10:**
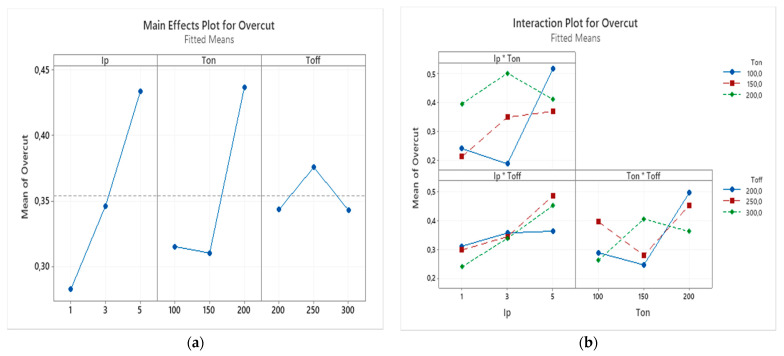
(**a**) The main effects plot and (**b**) the interaction effects plot for the overcut.

The interaction effects and main effects of process parameters of EDM of nonconductive ATZ on overcut were studied by conducting variance analysis by plotting these effects. In the Figure 10a the main effect plot for the overcut can be observed. For each parameter level, the means of overcut can be seen on this plot. Based on this plot, increasing the peak current raised the overcut. The increase in current resulted in the increase of MRR which in turn resulted in the increase of overcut. The effect of pulse on time on the overcut is not significant until 150 µs but is found to increase at 200 µs. The overcut increased slightly then decreased with the increase of pulse interval, as can be seen from Figure 10a.

Figure 10b shows the interaction effect plot for the overcut. Based on the interaction plot it can be observed that interaction effect between peak current and pulse off time is not very significant, as the lines are almost parallel. Although the interaction effect between pulse on time and pulse off time exhibits some variation in results, non-parallel lines indicate existence of interaction between these two parameters on the overcut. As it can be seen from Figure 9b, the peak current and pulse off time interaction effect have a significant influence on the overcut.

#### 3.3.2. Analysis of Circularity Error

**Figure 11:**
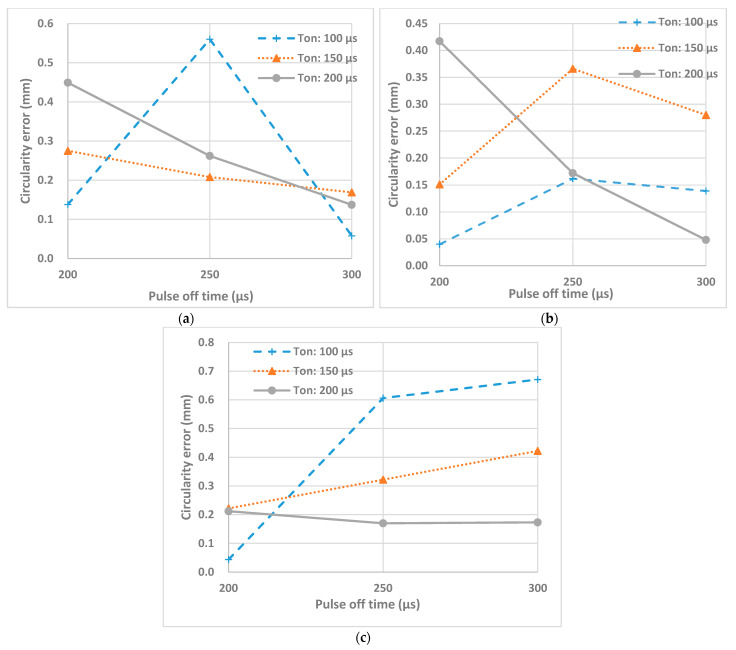
Effects of T_on_ and T_off_ on circularity error at peak currents (**a**) 1 A, (**b**) 3 A, (**c**) 5 A.

In this study, the circularity error of machined holes was measured in terms of maximum variation of diameter, i.e., distance between the highest and lowest point at the rim of hole. Figure 11 shows effects of pulse on time and pulse off time at different peak currents on the circularity of machined holes. It can be observed that at higher peak currents circularity error increases, due to higher discharge energy which leads to larger crater sizes. The larger the crater sizes the rougher is the hole edge and the higher is the variation between the maximum and minimum point at the hole profile. However, the higher pulse on time and pulse off time help to decrease circularity error as it can be seen in case of 3 A and pulse on time of 200 µs and pulse off time of 300 µs and in case of 5 A and pulse on time of 200 µs and pulse off time of 300 µs. This is mainly due to the improved machining stability at a combined settings of pulse duration and pulser interval. Higher peak currents allow for removing more material, however, too short of a pulse on and pulse off time will result in rough machining. This leads to an uneven radial surface that is the cause of high circularity error.

**Figure 12:**
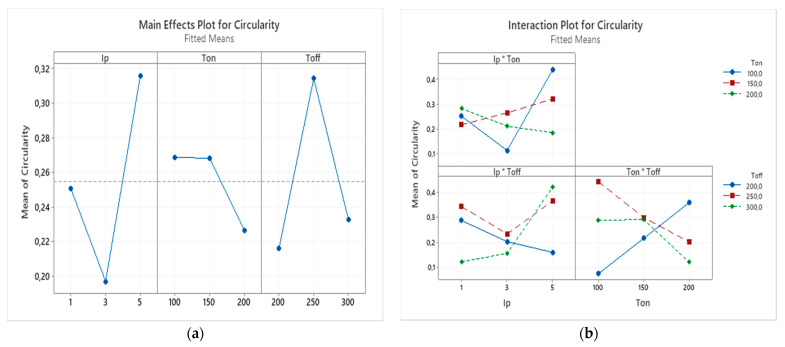
(**a**) The main effects and (**b**) the interaction effects plot for the circularity error.

The interaction effects and main effects of process parameters of EDM of nonconductive ZTA on circularity were studied by conducting variance analysis by plotting these effects. In the Figure 12a, the main effect plot for the circularity error can be observed. For each parameter level, the means of circularity error can be seen on this plot. Based on this plot, increasing the peak current from 1 to 3 A, resulted in the decrease of the circularity, which is favorable, however, increasing it from 3 to 5 A resulted the increase of the circularity error which is expected since increase in current leads to increase of crater radius and decrease in uniformity of machining. The increase of pulse on time showed only a slight decrease of circularity error. Pulse off time increase shows first sharp increase and after reaching 250 µs shows sharp decrease. The main effect plots using variance analysis does not indicate specific trends on effect of parameters on the circularity error of the machined holes during EDM of non-conductive ZTA ceramics.

Figure 12b shows the interaction effect plot for the circularity error. From this plot, it can be concluded that interaction effect between peak current and pulse on time and interaction effect between peak current and pulse off time are not significant, except for the 100 µm. The most interaction effect that carries significance for the circularity error is between pulse on time and pulse off time, which again prove the concept discussed earlier that a combination of optimum pulse on and off time are required for stable EDM operation and generating smooth surface at the rim of machined holes.

#### 3.3.3. Analysis of Taper

**Figure 13:**
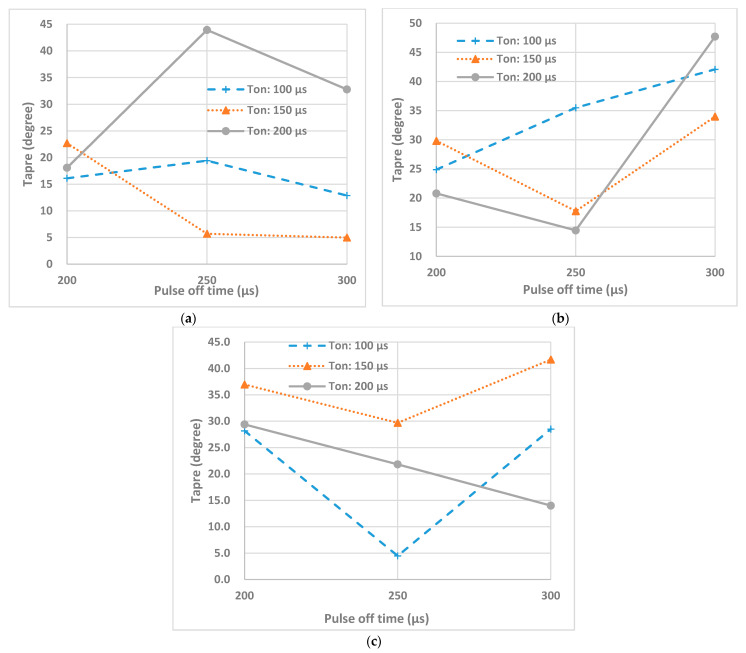
Effects of T_on_ and T_off_ on taper at peak currents (**a**) 1 A, (**b**) 3 A, (**c**) 5 A.

Figure 13 presents effects of pulse on time and pulse off time at different peak currents on the taper of machined holes. As the machining begins on the surface of conductive layer, the sparks are more intense and regular, thus leading to a higher radial overcut. As the machining continues and reaches the region of ceramics, discharge intensity decreases since sparks occurs only on the areas where carbon is deposited. Based on Figure 13, it can be suggested that pulse off time value of 250 µs is the point of change for machining at high enough pulse on time. For instance, the case with pulse on time of 200 µs for machining at 1 A and case with pulse on time of 150 µs and 200 µs for machining at 3 A and case with pulse on time of 150 µs for machining at 5 A, all show the change of trend either from increasing to decreasing or reverse. This may indicate that a pulse off time value of 250 µs is critical value for machining with these parameters for taper.

**Figure 14:**
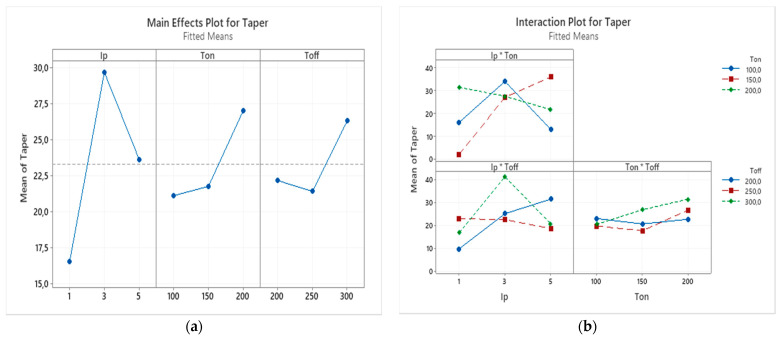
(**a**) The main effects and (**b**) the interaction effects plot for the taper.

The interaction effects and main effects of process parameters of EDM of nonconductive ZTA on taper were studied by conducting variance analysis by plotting these effects. In the Figure 14a the main effect plot for the taper can be observed. For each parameter level, the means of taper can be seen on this plot. Based on this plot, increasing the peak current from 1 to 3 A results in a sharp increase, however, further increase leads to a reduction of taper. The trend for pulse on time and pulse off time is similar. The increase in those parameters lead to a slight increase of the taper.

Figure 14b shows the interaction effect plot for the taper. From Figure 14b, it can be seen the interactions of pulse on time and pulse off time are presented by mostly parallel lines indicating no significant effect of this interaction on the taper of machined holes. However, based on the plot, it can be said that the most significant interaction effect for taper among different combinations is between peak current and pulse on time.

## 4. Conclusions

This study investigates the material removal mechanism and effects of machining parameters on the material removal rate and dimensional and profile accuracies of machined blind holes during EDM of Zirconia-toughened Alumina (ZTA). The following conclusions can be drawn from the experimental investigation:

- The assistive electrode comprised of multiple layers of conductive coatings along with nanoparticles was found to be effective in the successful EDM of electrically non-conductive ZTA ceramics. Although ZTA is non-conductive and EDM requires the material to be electrically conductive, experiments show a successful machining of the workpiece by the demonstration of both blind and through holes. The progression of material removal process depends on the modified machined surface, where electrically conductive material from the assistive electrode, tool electrode, and hydrocarbon dielectric oil migrates to the machined surface. The strong presence of carbon on machined surface indicates possible formation of carbides, thus facilitating the continuous sparking and therefore removal of materials.
- The dominating material removal mechanisms observed in this study during EDM of non-conductive ceramics ZTA are thermal cracking and thermal spalling. Thermal spalling process starts with thermal cracking followed by the peeling of conductive layers that are formed during the machining process as a result of migration of electrically conductive materials and resulting surface composition. The machined surfaces for all machining parameters were found to be rougher than traditional EDM machined metal surfaces indicating different material removal mechanisms. Due to the presence of melted and re-solidified materials and cracks, it can be suggested, that the material removal mechanism also includes melting and evaporation along with thermal crack and spalling.
- The increase in peak current resulted in the increase of discharge energy, which in turn raised the MRR. Increased pulse duration (T_on_) caused higher MRR but also resulted in redeposition of thick layer. Increased pulse interval (T_off_) also facilitates the total removal of debris from the machined zone. However, an optimum T_off_ value is critical for machining of nonconductive materials, as complete removal of conductive debris particles may slower the intensity of sparking on the machined surface. Therefore, a suitable combination of pulse on time and pulse off time needs to be identified for stable and continuous material removal during EDM of non-conductive ceramics.
- For machining at 1 A peak current, increasing the pulse off time at pulse on time of 150 µs leads to a rapid decrease of the MRR. For the machining at 3 A, rapid decrease of the MRR can be observed at pulse on time of 200 µs.
- Increasing the pulse off time generally leads to a decrease of overcut. However, there are cases with the increase of overcut for increasing pulse off time at pulse on time of 150 µs and peak currents of 3 A and 5 A. Based on the main effects plot, the increase in the current results in the increase of MRR which in turn resulted in the increase of overcut. It was also concluded that interaction effect of peak current and pulse on time is significant for overcut.
- Increasing the peak current from 1 to 3 A resulted in the decrease of the circularity error, however increasing it from 3 to 5 A resulted in the increase of the circularity error which is expected since an increase in the current leads to increase of crater radius and decrease in uniformity of machining. Increase of pulse on time showed only a slight decrease of circularity. The pulse off time increase reveals sharp increase initially and after reaching 250 µs it demonstrates sharp decrease. At higher peak currents, circularity, i.e., irregularity at the edge of the machined holes, increases, due to higher discharge energy which leads to larger craters sizes. However higher pulse on time and pulse off time help to decrease circularity. Higher peak currents allow removing more material, however, too short pulse on and pulse off time result in rough machining. This leads to uneven radial surface causing high circularity error.
- Although increasing the peak current from 1 to 3 A results in a sharp increase however further increase leads to a reduction of taper. The trend for pulse on time and pulse off time is similar. Increase in those parameters lead to a slight increase of taper. It can be noticed that the most significant interaction effect for taper among different combinations is between peak current and pulse on time.
- As the machining begins on the surface of conductive layer, the sparks are more intense and regular, thus leading to higher radial overcut. As the machining continues and reaches the region of ceramics, discharge intensity decreases since sparks occurs only in the areas where electrically conductive materials are deposited. The most significant interaction effect for taper was found to be between peak current and pulse on time.
- Finally, this study demonstrates that using the proposed layered assistive electrode method, successful machining of through and blind holes can be achieved on the electrically non-conductive ceramic ZTA using the EDM process. This study provides insights into the material removal mechanisms and effects of important machining parameters on the EDM performance, thus, will help future researchers to attempt further investigation into the EDM of non-conductive ceramics.

## Figures and Tables

**Table 1:** Summary of ceramics fabrication techniques.

Fabrication Method	Process	Pros	Cons	References
Chemical Machining	Manufacturing process where etchants are used to attack workpiece material and small amounts of material are removed. Since this method is suitable only for shallow material removal, structural features such as deep cavities, sharp corners and porous workpieces are not easily machined.	Stress absence on workpiece; tool and capital costs are low; weight is reduced near complex contours	Chemical’s handling and disposal; difficult to process porous materials; surface defects; cuts are shallow	[3,4]
Plasma Machining	Material is machined by using ionized gas at high temperatures. This results in good surface finish and small kerf widths.	Independent of hardness; less operation energy is required [5]; capital costs are low	Poor accuracy (±0.762–1.524 mm); difficult to process thick materials; edge quality is poor; significant heat distortion	[5,6]
Laser Machining	Machining process where optical energy of high density is incident on material surface. Material ablation occurs through melting, evaporation, decomposition, and material expulsion.	Independent of hardness and electrical conductivity; acceptable edge quality and dimensional integrity	Beam geometry causes inaccuracies in geometry; recast layer and heat affected zone are significant; difficult to machine thick materials; surface quality is poor (macro-cracks, surface damage) [7]	[7,8,9]
Ultrasonic Machining	Material removal occurs by abrasive particles which are vibrated ultrasonically. The tool is vibrated at high frequency along its longitudinal axis.	Low thermal effect; surface quality is good	High cost of abrasive particles; material removal rate is low	[10,11,12]
Electron Beam Machining	In this process heat is generated by collision of workpiece surface and high-speed electrons. Because deflection coil rapidly positions electron beam, high-speed machining is possible	Independent of hardness, reflectivity, and ductility; high material removal rate; good accuracy and repeatability	Recast layer exists; Auxiliary backing material is needed; high equipment cost; production time is long	[13,14,15]
Electro-chemical Machining	This technique of machining is suitable for producing complex cavities in materials of high strength. An electrolyte is used to erode material from surface or sharp profiles. Hence, it cannot be used to produce sharp corners.	Negligible tool wear; intricate shapes can be manufactured; high resolution [16]	Low efficiency; storage and handling of electrolyte is difficult; poor accuracy due to side machining effect	[17,18,19]
Abrasive Waterjet Machining	In this method, a blast of water stream strikes the surface of the workpiece and leads to erosive wear.	High-speed cutting; operating and capital costs are low [20]; absence of thermal effect; low tool wear and machining time	Kerf formation due to hydrodynamic forces; abrasive is needed; low surface quality; difficult to manufacture intricate shapes and to cut thick workpieces [21]	[22,23,24]
Grinding	In this method, rotating grinding wheel containing abrasive grains remove material due to relative motion.	Very fine chip size is possible, very high shape accuracy and dimensional precision	Higher specific energy, cracked surface, sintered ceramic difficult to machine, time consuming and expensive	[25]
Direct Additive Manufacturing	“Single-step” or “multi-step” AM process where layer by layer	Mold-free process, cost reduction, short lead time, design freedom	High melting points, low tolerance to processing defects, limited choice of materials, excessive surface roughness, lumps, internal porosity, and surface micro- and macro-cracks	[26]

**Table 2:** Properties of Zirconia toughened Aluminum (ZTA).

Parameter	Al_2_O_3_ (%)	ZrO_2_ + Y_2_O_3_ + CeO_2_+ La_2_O_3_ (%)	Density (g/cm^3^)	Compressive Strength (MPa)	Fracture Toughness (Indentation Method) Mpa.m^1/2^	Vickers Hardness (Kgf/mm^2^)	Electrical Resistivity (Ohms-cm)^−1^
Amount	75.68	23.62	4.14	492	4.6	1426	10^−14^ (25 °C)

**Table 3:** Specifications of Excetek ed 400 Die- Sinker electro discharge machining (EDM).

Criteria	C Codes	Pulse on Time	Pulse off Time	Peak Current	HV Discharge	LV Discharge	Discharge Time	Gap Voltage
Description	Predefined 2000 sets	0–2000 µs	0–4000 µs	0–288 amps	140 V–235 V DC	0.4–1.8 Amps	0.1–99.9 s	20–120 V

**Table 4:** Parameters and results of the experiments on ZTA at 80 V.

No.	I_p_ (A)	T_on_ (µs)	T_off_ (µs)	Diameter (mm)	Machining Time (s)	Depth (mm)	Overcut (mm)	Circularity (mm)	Taper (degree)	MRR (mm^3^/s)
1	1	100	200	2.373	1145	0.870	0.237	0.138	16.111	2.699 ×10 ^−3^
2	1	100	250	2.520	1775	1.332	0.310	0.560	19.412	2.522 ×10 ^−3^
3	1	100	300	2.250	5900	1.550	0.175	0.058	12.883	7.504 ×10 ^−4^
4	1	150	200	2.348	4552	2.588	0.224	0.275	22.701	9.847 ×10 ^−3^
5	1	150	250	2.326	1418	1.175	0.213	0.208	5.703	3.178 ×10 ^−3^
6	1	150	300	2.298	3185	1.882	0.199	0.169	4.996	2.116 ×10 ^−3^
7	1	200	200	2.845	1411	0.807	0.473	0.449	18.080	3.003 ×10 ^−3^
8	1	200	250	2.641	1021	0.684	0.371	0.262	43.922	2.145 ×10 ^−3^
9	1	200	300	2.590	1152	0.948	0.345	0.137	32.761	2.615 ×10 ^−3^
10	5	100	200	2.724	1395	1.552	0.412	0.044	28.175	3.331 ×10 ^−3^
11	5	100	250	3.272	3900	1.602	0.686	0.606	4.484	3.195 ×10 ^−3^
12	5	100	300	2.816	2628	2.787	0.458	0.671	28.506	2.641 ×10 ^−2^
13	5	150	200	2.490	823	1.080	0.295	0.222	36.948	3.127 ×10 ^−3^
14	5	150	250	2.517	727	1.227	0.309	0.322	29.692	4.595 ×10 ^−3^
15	5	150	300	2.913	732	0.977	0.507	0.422	41.683	4.640 ×10 ^−3^
16	5	200	200	2.665	729	1.279	0.383	0.212	29.396	5.450 ×10 ^−3^
17	5	200	250	2.824	403	0.878	0.462	0.170	21.815	1.053 ×10 ^−2^
18	5	200	300	2.679	715	1.490	0.390	0.173	13.997	8.790 ×10 ^−3^
19	3	100	200	2.336	690	0.771	0.218	0.040	24.900	3.468 ×10 ^−3^
20	3	100	250	2.280	743	0.942	0.190	0.162	35.505	2.723 ×10 ^−3^
21	3	100	300	2.207	723	0.863	0.154	0.139	42.094	2.101 ×10 ^−3^
22	3	150	200	2.341	763	0.808	0.220	0.151	29.822	2.992 ×10 ^−3^
23	3	150	250	2.534	860	1.140	0.317	0.366	17.772	4.943 ×10 ^−3^
24	3	150	300	2.922	684	1.024	0.511	0.280	33.986	6.045 ×10 ^−3^
25	3	200	200	3.165	729	1.452	0.632	0.417	20.800	1.084 ×10 ^−2^
26	3	200	250	2.952	848	1.388	0.526	0.172	14.456	8.703 ×10 ^−3^
27	3	200	300	2.601	728	0.604	0.351	0.048	47.696	2.542 ×10 ^−3^

**Table 5:** Measurement of average lengths of microcracks on the machined ceramic surface at different machining conditions.

Voltage (V)	Current (A)	Pulse on Time, T_on_ (μs)	Pulse off Time, T_off_ (μs)	Crack Length (μm)	Average Crack Length (μm)
80	1	100	200	76.532	54.128
				63.322	
				40.198	
				53.105	
				37.484	
80	1	100	250	80.726	71.231
				69.796	
				63.172	
80	1	150	250	89.038	82.900
				76.819	
				78.511	
				87.232	
80	1	150	300	302.414	249.657
				206.321	
				240.235	
